# Thermal Regeneration of Spent Sand with Furfuryl Binder from an Ecological and Economic Point of View

**DOI:** 10.3390/ma16227102

**Published:** 2023-11-09

**Authors:** Mariusz Łucarz, Michał Dereń

**Affiliations:** Faculty of Foundry Engineering, AGH University of Science and Technology, Reymonta 23 St., 30-059 Krakow, Poland

**Keywords:** thermal regeneration, spent sand, BTEX analysis, energy consumption, equipment

## Abstract

The recovery of the grain matrix from spent moulding sand is a constant challenge in making the best possible use of the deposits of quartz sand material, as well as in protecting them. In the case of spent sand with organic binders, the best method to recover the grain matrix is thermal regeneration. However, this method is expensive and requires adequate attention to the emission of harmful compounds into the atmosphere. This paper presents a new concept for implementing the thermal regeneration process. A suitable regeneration temperature was adopted for the furfuryl binder moulding sand, and a change in the design of the device was introduced in the area of the utilisation of gases generated during the combustion of the spent binder. To confirm the assumptions made, and to assess the appropriate suitability of the material recovered, the technological parameters of the material obtained were verified, namely, ignition losses, sieve analysis, bending strength, and pH value. The consumption of media for the process was also analysed from an economic point of view, as well as the emission of BTEX (a mixture of volatile aromatic hydrocarbons-benzene, toluene and three isomers of xylene) gases under different conditions of the process. On the basis of the research conducted, it was concluded that lowering the regeneration temperature of regeneration does not adversely affect the technological parameters of the moulding sand on the regenerate matrix. Changing the design of the regenerator does not result in increased emissions of harmful substances to the environment. Studies indicate that the appropriate setting of thermal regeneration parameters and the optimal design of the employed equipment are favourable factors in reducing the cost of the process while not compromising the quality of the moulding sand and the environmental impact.

## 1. Introduction

With increasing requirements for environmental protection, the improvement of working conditions and safety, and a reduction in the industry’s impact on the ecosystem, foundries are looking for technological solutions to increase the quality of the castings produced while limiting the emission of harmful substances and reducing the need for natural raw materials. This is not only due to legal considerations but also the criteria of foundry customers, which relate not only to the quality of the casting but also to the foundry’s environmental policy. Thus, customers are moving towards sustainable development, forcing foundries to improve their technological processes in terms of the environment and occupational safety and to introduce additional requirements in this respect.

Various types of moulding sand reclamation are used in the foundry industry [1], which, in addition to dust, generates significant amounts of other waste after the casting production process. There are three basic grain matrix recovery methods that are used: wet in water is used mainly for bentonite and water glass moulding sands; mechanical is for all types of spent moulding and core sands; thermal is for burnt binders. Due to the costs of infrastructure and water and energy consumption, wet regeneration is rarely used [1,2,3,4]. Depending on the type of binder material, the other methods are applied using different equipment. The process of mechanical regeneration is presented by the authors in publications [5,6,7,8,9,10,11,12,13,14,15,16,17,18,19,20,21,22,23,24,25,26,27,28,29,30]. However, the issues of removing spent organic binders via thermal regeneration are addressed in publications [31,32,33,34,35,36,37,38,39,40,41]. The issues of the cost of the mechanical regeneration process and the promotion of this method for the recovery of waste foundry sand (WFS) are presented in publications [9,42].

Among the various methods of recovering the sand matrix, thermal reclamation deserves special attention [40,41]. This process is perceived as an effective way of obtaining the best quality regenerate when organic binders are used in foundries; however, at the same time, it is one of the most expensive regeneration methods [1,43]. This is due not only to the energy input required to fire the binder but also to the cooling process and the need to limit the emission of gases generated during the combustion of the resin (in the regenerator or in the reactor, as well as in the use of filter systems). Due to the significant increases in the price of waste disposal of spent sand [43] from foundries, the thermal regeneration method is gaining importance. An additional advantage is also the cost of the process itself: With current prices for the purchase of sand matrix, its transport to the foundry and the spent sand to the landfill, landfill fees, and the regeneration yield after the regeneration process, the thermal method becomes even cheaper than the mechanical method, taking into account the longer period of application of the thermal sand matrix recovery technology [43]. The amount of spent sand resulting from mechanical regeneration is much higher than in the case of thermal regeneration, which affects the cost of storage and increases the need for fresh sand matrix (to replenish the amount of stock in the foundry circuit). Thermal regeneration can also be used as one of the regeneration steps for modern and environmentally friendly inorganic binder sand, especially as the use of renewable energy sources in the industry can be increasingly observed, further influencing the rationale for the development of this regeneration method. When considering the ecological and environmental aspects, the advantage of wasted sand created after the thermal regeneration process is its lower toxicity regarding the leaching of chemical compounds during storage in industrial landfills [44].

Global manufacturers of thermal regenerators often use universal parameters for the regeneration process in their equipment (e.g., temperature and time), aiming to carry out all process steps such as binder burning and gas reduction in a single chamber [1]. The recommended temperature for the thermal regeneration process, according to the manufacturers of the respective equipment, is applicable to most organic binders commonly used in the foundry industry, regardless of what the burning temperature limit is for the resin used in a given foundry. Changing the temperature to an adequate temperature for the spent binder reduces the energy requirement and affects the life of the refractory lining inside the burning chamber in the regenerator [43].

Due to the growing importance of thermal regeneration in the foundry industry, it was decided to further improve it. The focus was on changing the parameters of the thermal regeneration process. An additional gas afterburning chamber was used in the process, which, in order to perform its function of limiting flue gas emissions correctly, must create two fundamental conditions: the temperature should be in the range of 800–1200 °C, and the residence time of the gases in this temperature range should be, at minimum, 3 s depending on the type of chemical compounds [43,44,45,46,47,48]. The designs are mostly based on the generation of high temperatures through the use of gas burners [43,46]. Another method [49] works on the basis of microwave energy and suitable ceramic elements.

Spent moulding sand is also used in road construction and the building materials industry [50,51]. This requires the proper environmental identification of these sands [52].

In terms of reducing the wear of natural sands due to crushing and abrasion and the formation of large amounts of dust, attempts are being made to introduce synthetic sands [53].

A new issue in the area of recycling spent moulding sand and core sand is the regeneration of 3D printing waste. More and more attempts are being made to bind sands with various binders using this method [8,54,55].

In this study, the possibility of recuperation was used to reduce the energy demand during the process.

## 2. Materials, Research Methods, and Experiment

### 2.1. Material

The tests were carried out on two types of moulding sand. The first (type A) moulding sand was prepared in a mixer for continuous mixing, while the second (type B) moulding sand was made using a 3D printer (EXOne S-Max, EXOne Operating, LLC, North Huntingdon, PA, USA). The parameters of the initial grain matrices are shown in Table 1.

The sand grains for 3D printing are much smaller than the grain matrix used in standard moulding or core sand preparation.

**Figure 1:**
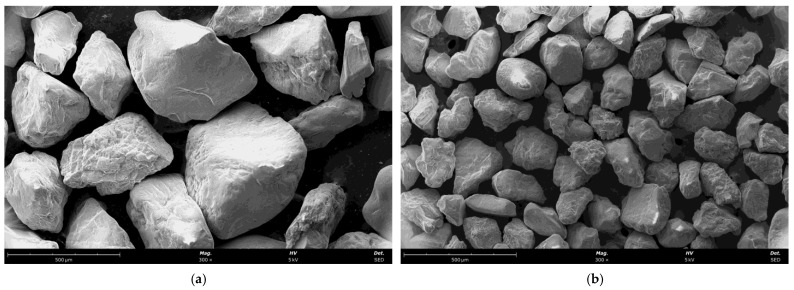
Initial grain matrices used in the study: (**a**) moulding sand type A, mag. ×300, (**b**) moulding sand type B, mag. ×300.

Figure 1 shows SEM (scanning electron microscope) images of the grain matrices used in the manufacturing.

Table 2 shows the compositions of the binder used in the tests.

The resin used in the 3D printer is furfuryl resin, cured with p-toluene sulphonic acid. The binder used in 3D printing technology does not differ in its main chemical composition from the classic binder but only contains additives that affect the viscosity of the resin. This parameter is important because of the very high precision required from the resin-dispensing head during the sand-layer printing process.

The sand matrix obtained from the moulding sand after thermal regeneration, which was carried out at a given temperature, was used to make moulding sand of the same composition to assess the quality of the moulding sand and prepare the moulds for casting.

### 2.2. Testing Methods

Table 3 shows the methods used to evaluate the regeneration of spent sand with synthetic resin and other types of spent sand, taking into account the hierarchy of their use [1,8,56].

As can be seen in Table 3, five methods are relevant to evaluate the quality of the regenerate extracted from the spent furan (synthetic) binder. Individual test methods are characterised below.

The measurement of the ignition losses was carried out in accordance with BN 83/4021 and was carried out as follows: a 50 g sample weighed on a Radwag MA 110.R balance (RADWAG Wagi Elektroniczne, Radom, Poland) was inserted into a ceramic crucible. Three samples were weighed for each ignition loss analysis. The samples were then placed in a Remix KRE-11/8 roasting furnace (Remix S.A., Świebodzin, Poland), with a roasting temperature of 900 °C and a holding time of 1 h in the furnace. After roasting, the samples were allowed to cool in a desiccator. After reaching room temperature, the samples were again weighed on a Radwag MA 110.R balance with an accuracy of 0.01 g. Based on the difference in the obtained weights of the samples before and after roasting, the average value of the ignition losses was calculated.

**Figure 2:**
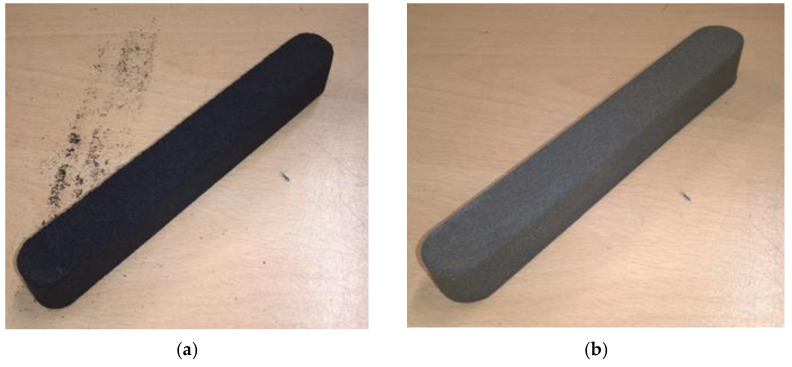
Bending strength test specimens: (**a**) shape made from type A moulding sand prepared in a mixer for continuous mixing; (**b**) shape made from type B moulding sand prepared with a 3D printer.

The moulding sand test was carried out on an LRu-2e device (Multiserw-Morek, Marcyporęba, Poland) according to the requirements of the PN-83/U-11073 standard [57]. The bending strength test specimen is shown in Figure 2a (type A moulding sand) and Figure 2b (type B moulding sand). In both cases, the dimensions of the sample were 22.36 mm × 22.36 mm × 172 mm.

For each type of moulding sand (type A and type B), samples were made as follows:

- For the standard technology using a mixer for continuous mixing, the execution of the sample consisted of backfilling the model, reflecting its shape with the sand prepared in the mixer for continuous mixing and compaction on the LUZ-2e standardised specimen vibration device from Multiserw-Morek (Marcyporęba, Poland) (Figure 2a);
- For 3D printing technology (type B moulding sand), the specimens were made using a 3D printer according to the requirements of the standard for testing the bending strength of the moulding sand (Figure 2b).

Each type of specimen (A and B) was measured at appropriate time intervals from the completion of the filling of the mould for compacting or printing the specimen, i.e., after 1, 2, 4, and 24 h. After the strength samples were prepared, they were placed in a room with stable temperature and humidity conditions. The results presented in this publication are the average of three measurements.

The pH of the moulding sand was carried out using a Metrohm 916 Ti-Touch pH meter (Metrohom AG, Herisau, Switzerland). The measurement process consisted of measuring a 25 g weight and placing it in a laboratory vessel with 25 mL of distilled water and a magnetic mixer. The mixer was then run for 5 s. After the mixing was complete, the mixture was left for 15 min; then, stirring was carried out again, and the pH of the solution was measured with a pH meter. For each solution obtained, 3 measurements were taken at 1 min intervals, and the average value was calculated from these.

Sieve analysis was performed on an LPzE-2e laboratory shaker from Multiserw-Morek with a set of metal sieves that conform to DIN ISO 3310-1 [58]. The mesh sizes on the sieves were as follows: 1.4; 1.0; 0.71; 0.5; 0.355; 0.25; 0.18; 0.125; 0.09; 0.063 mm. The measurement consisted of placing a 50 g weight on a laboratory shaker and running the vibration for 15 min. After this time, a sample of the sand deposited on each sieve was collected and weighed. The main fraction of sand was determined by the largest amount of sand collected on three adjacent sieves. The results presented in the paper are the average of the two measurements.

The study of the surface morphology of the sand matrix and its chemical composition after the thermal regeneration process was carried out on a TermoScientific Phenom ProX scanning microscope (TermoScientific, Waltham, MA, USA). The image was taken using the Back Scatter Electron (BSE) function (Oxford instruments, Abingdon, UK) and the chemical composition with the energy dispersive spectroscopy (EDS) attachment. Before measurement, the sample was dried in an oven at 50 °C for 1 h. After this time, it was placed in a desiccator to cool to room temperature. The sand samples were placed on carbon disks, which were attached to special stands with a pin to attach to the microscope working handle.

The most important element of the research carried out was the analysis of the gases emitted during the regeneration of the spent moulding sand. The concentration of the evolved gases was measured downstream of the afterburning chamber and upstream of the filter system. A spigot with a measuring probe was installed in the extraction pipeline, which was inserted up to ½ of its diameter. The gases were extracted from the pipeline using an ASP 3II aspirator manufactured by LAT (LAT Sp. z o.o., Katowice, Poland) with a reading accuracy of 0.1 dm^3^ and a display resolution of 0.1 dm^3^/h. The gas samples collected in this way were analysed in a gas chromatograph with an Agilent 7820A flame ionisation detector (GC-FID) made by Agilent Technologies (Santa Clara, CA, USA).

The above method of measurement was performed analogously for each set temperature in the regenerator chamber with the afterburning chamber switched on and off. Samples were taken 30 min after a stable temperature had been reached in the regenerator chamber and, simultaneously, the first-stage regenerant intended for thermal regeneration had started (for both type A and B sand). The gas concentration value obtained is the arithmetic mean of the two measurements.

### 2.3. Experiment

In this study, an attempt was made to reduce the costs of the thermal regeneration of spent furan binder moulding sand by lowering the regeneration temperature in an industrial plant and reducing the amount of energy media (gas and electricity) consumed. At the same time, the measures taken did not increase the adverse impact on the environment through the resulting gas emissions when the spent binder at a lower temperature.

**Figure 3:**
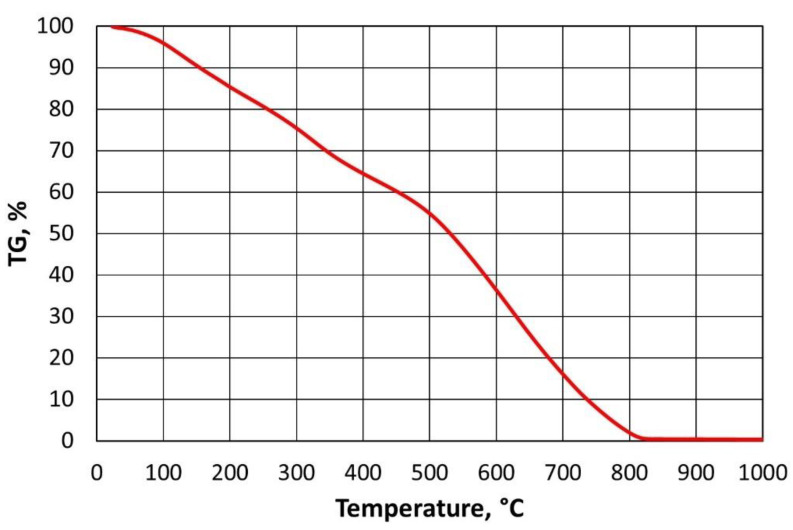
TG curves for furan binder samples in an oxygen atmosphere.

**Figure 4:**
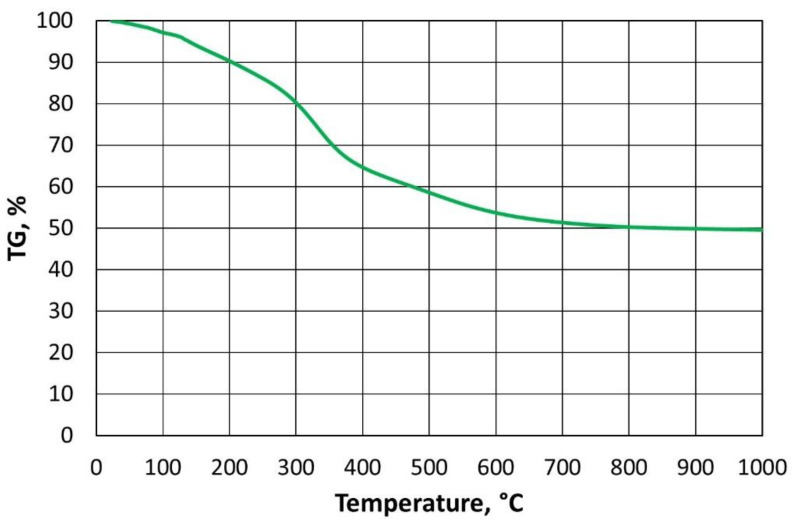
TG curves for furan binder samples in an oxygen-free atmosphere.

In order to reduce the cost of thermal regeneration, it is important to determine the required thermal regeneration temperature, i.e., one that is sufficient enough to burn the spent binder and requires the lowest possible energy input. The paper [43] presents a suitable laboratory method to determine such a temperature for a furan binder. The procedure developed and described in detail to determine the required regeneration temperature for a given organic binder in [44] is based on thermogravimetric analysis performed in air and in an oxygen-free atmosphere. A characteristic feature of the TG (thermogavimetric analysis) plot obtained (Figure 3) for the exemplary furan binder is that, once the specified temperature is exceeded, a rectilinear section of the mass change of the binder sample tested is noticeable (temperature range 477 °C to 789 °C). At lower temperatures, the bonded resin was degraded (curvilinear percentage course of mass change), while the proportional mass loss should be taken as a successive combustion of the binder, which varies by less than 0.10% from 812 °C onwards. A similar relationship can be seen in Figure 4, performing thermogravimetric analysis under anaerobic conditions (argon atmosphere). As the temperature in the analyser chamber increases from a certain value, the mass of the test sample changes linearly (temperature range from 845 °C to 1000 °C).

If we compare the data in Figure 3 with the results in Figure 4, it can be concluded that, over a certain temperature range, furan binder breakdown (degradation) occurs regardless of the atmosphere in which the process is carried out. Changes in TG values are not identical but follow a similar pattern. Only above a temperature of 500 °C can the atmosphere in which the test is carried out begin to play an important role. In the presence of atmospheric oxygen, the sample burns (destroys), hence the almost proportional weight loss of the furan binder. In contrast, the second sample, in the absence of oxygen in the analyser’s working space (Figure 4), has not fully decomposed by 1000 °C, leaving approximately 49.54% of the sample mass containing carbonised carbon.

If straight lines are drawn through the rectilinear sections of the curves obtained, their point of intersection can be taken as the minimum temperature required for the thermal regeneration of the binder under test; from this limiting temperature, the residual decomposition products (carbonised carbon) resulting from the degradation of the binder are successively burnt off. From an analysis of Figure 3, it can be seen that the complete combustion process occurred at a temperature of approximately 812 °C; however, it should be assumed (noted) that it is not the temperature, which builds up at a rate of 10 °C/min in the NETZSCH STA 449 F3 Jupiter^®^ thermal analyser (Erich NETZSCH GmbH & Co. Holding KG, Selb, Germany), that plays the main role, but rather the time required for the complete combustion (oxidation) of the sample of the binder under test.

To determine the minimum required regeneration temperature, a system of linear Equation (1) was introduced, which, in general, takes the following form [43]:

(1) $$\{\begin{array}{c} TG_{air}=a_{air}\times T+b_{air}\\ TG_{Ar}=a_{Ar}\times T+b_{Ar} \end{array}$$

The following mathematical procedure was adopted for the determination of the equations. For thermogravimetric analysis in the air and argon atmosphere, the difference between the individual percentage mass losses was calculated for a constant temperature change between the individual values (2):

(2) $$\Delta TG=TG_{i}-TG_{i-1}$$

For the analysis carried out in air, the maximum value of Δ*TG_max_* is above 450 °C, which corresponds to the greatest slope of the analysed curve to the 0X axis (greatest angle between mass loss and constant temperature step). All percentage mass losses that are greater than half the Δ*TG_max_* value were taken as proportional changes in the graph.

For tests in an argon atmosphere, a minimum value of Δ*TG_min_* above 450 °C was set. In this case, it was estimated that a proportional change in the thermal analysis curve would occur when the percentage losses were no greater than twice Δ*TG_min_*, which corresponds to the smallest slopes of the individual calculated values on the 0X axis.

The point of the intersection of the determined linear functions should be taken as the value sought (3):

(3) $$TG_{air}f\left(T\right)=TG_{Ar}f\left(T\right)$$

**Figure 5:**
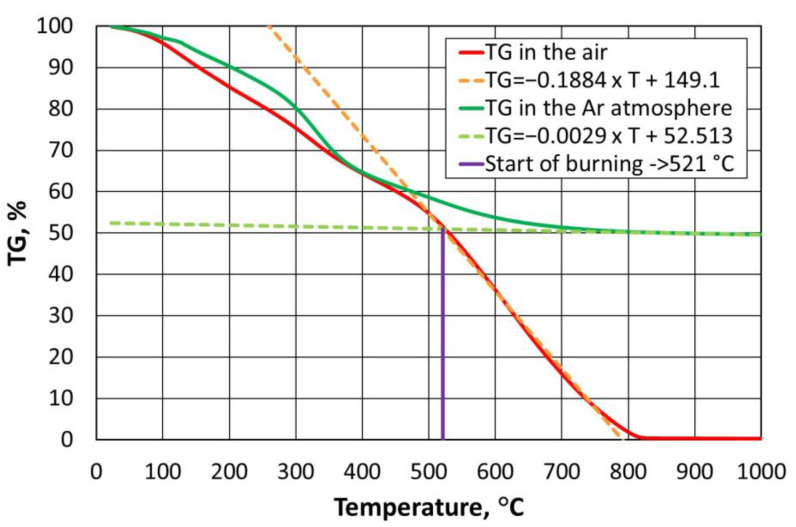
Selection of combustion temperature for furan binder.

Linear functions were selected for the range of data for the furan binder varying along rectilinear sections, and the resulting equations are shown in Figure 5.

In the case of the furan binder, a system of the following equations was obtained:

(4) $$\{\begin{array}{c} TG_{air}=-0.1884\times T+149.1,\;\;\;\;\;\;\;\;\;\;\;\;\;\mathrm{where}\;R^{2}=0.9969\\ TG_{Ar}=-0.0029\times T+52.513,\mathrm{where}\;R^{2}=0.996 \end{array}$$

The solution to this system of equations is a temperature of *T* = 521 °C. This is a sufficient temperature for the combustion (destruction) of the analysed example (furan binder) [43], given that there is sufficient time to carry out the process.

The regeneration of spent moulding or core sand with organic binders can be divided into two stages: stage I preliminary (mechanical) regeneration and stage II proper (thermal) regeneration.

**Figure 6:**
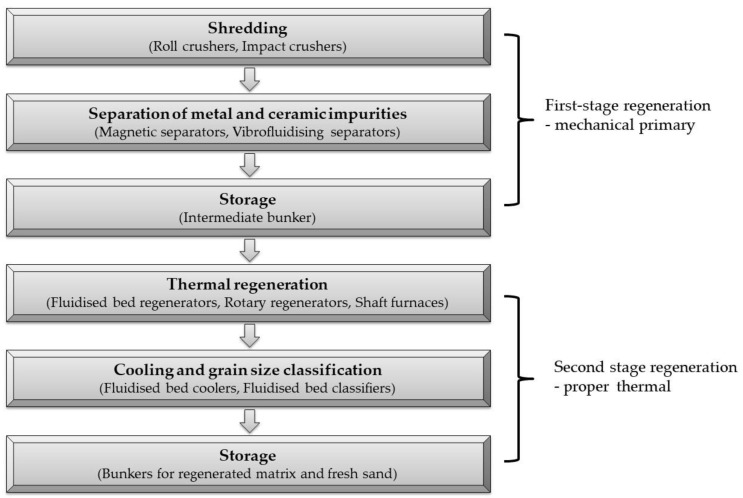
Generalised diagram of thermal regeneration activities and equipment.

The spent sand with organic binders created after the casting has been removed is usually in various states of decomposition. Some of the resulting waste is in a loose state and some is caked. To effectively perform the thermal regeneration process, it is subjected to crushing, that is, preregeneration (Figure 6) [59]. During the crushing of a mixture of bulk and clumped material, mechanical degradation occurs, which refers to macroscopic effects occurring in the polymers under stress. The reason for the reduction in strength is the inhomogeneity of the material, i.e., the presence of weakened areas in the material, which are the beginning of the destruction of the bridges formed from the binder and connecting the matrix grains. Weak areas include chain ends, aggregates of low-molecular-weight polymer fractions, inclusions of foreign bodies, and residues of monomer, solvent, hardener, etc. Significant stresses are concentrated at these sites, which are microcarbons [60].

The proper regeneration carried out in the thermal regenerator chamber is designed to realise four aspects of the process:

- Aeration of the bed via fluidisation mixing in order to burn the layer of thermally treated material uniformly as a result of the supply of oxygen needed for combustion;
- Burning the envelope of organic material from the surface of the matrix grains;
- Limiting the lifting of fine particles from the regenerator chamber into the chimney;
- Post-combustion of gases generated after the decomposition process of organic binders.

**Figure 7:**
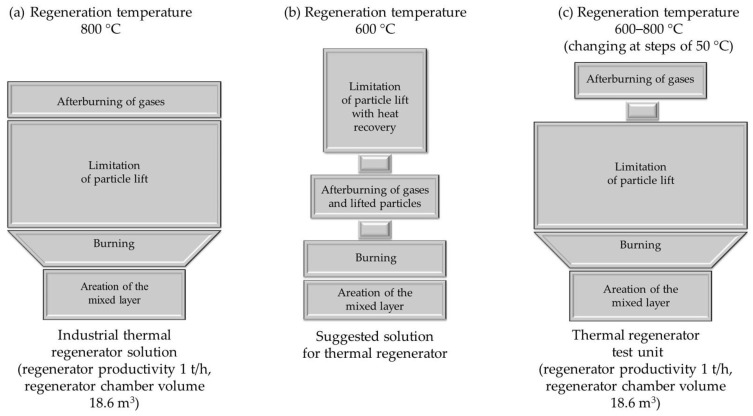
Schematic diagram of the thermal regenerator: (**a**) existing solution; (**b**) suggested solution; (**c**) developed test bench.

Schematically, the proper regeneration carried out in a thermal regenerator is shown in Figure 7a.

The realisation of these four stages of thermal regeneration requires a specific design of the regenerator chamber, which is characterised by a large height in relation to the bed layer of the regenerated used moulding or core sand.

A suitably large chamber height is needed because of the fluidised granular material of varying granularity. The height of the chamber, which is often extended to the top, is designed to slow down the velocity of the particles of varying granulation. In addition to fine matrix grains and dust, gases from the thermal decomposition of organic binders also move into the flue system. The requirement is that the flue gases are free of substances that have a negative impact on the environment. For this reason, in the thermal regenerator, high temperatures are generated throughout the chamber to burn off the gases generated during regeneration (temperature of more than 800 °C, heating time of 3 s), hence the use of additional burners in some furnace solutions.

Figure 7b presents a proposal for a different solution to the thermal regenerator based on existing designs and taking into account the phenomena observed during the thermal regeneration process tests carried out. In the suggested solution, the regeneration chamber is split into several modules in which the individual stages of thermal regeneration are separated.

In the first segment, regeneration is assumed to be carried out at the selected temperature for the organic binder in question (the temperature selection method for the furan binder is presented above). This part of the regenerator, as the main mixing and combustion chamber, can have a more compact structure and a smaller height, since its task is only to cause the thermal decomposition of the resin and the combustion of the carbon residue (carbonisation).

The task of the second member is to post-combust the resulting gases and uplifted dust. As outlined in [43,44,45,46,47], two basic conditions must be created in such an additional chamber of different design using generally available energy sources:

- The temperature, depending on the chemical compounds to be neutralised, must be in the range of 850–1200 °C;
- The flue gas must be exposed to the temperature for at least 3 s.

The third section can be used to capture the fine grains of the matrix in combination with a heat exchanger to heat the air used to fluidise the bed of regenerated sand, with simultaneous cooling of the exhaust gas prior to further purification in filter systems.

Under industrial conditions, it was not possible to interfere with a commercial organic binder sand regeneration unit. Therefore, in the course of the research, a cursory action was chosen, opting only to add, in addition to the standard thermal regenerator system, a flue gas afterburning chamber of our own design while reducing the temperature during regeneration, which is set at 800 °C in the device studied [1,20,61].

Figure 7c shows the idea of the test rig created.

**Figure 8:**
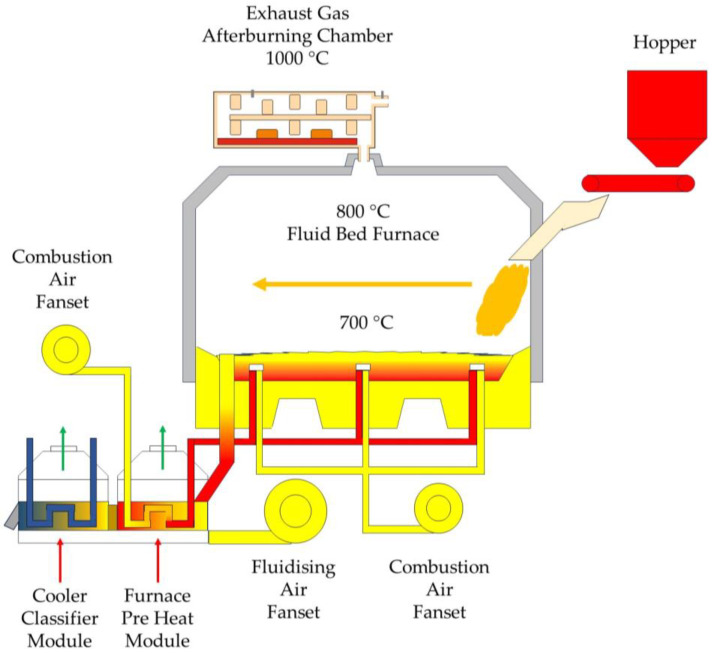
Explanatory diagram of the test stand.

**Figure 9:**
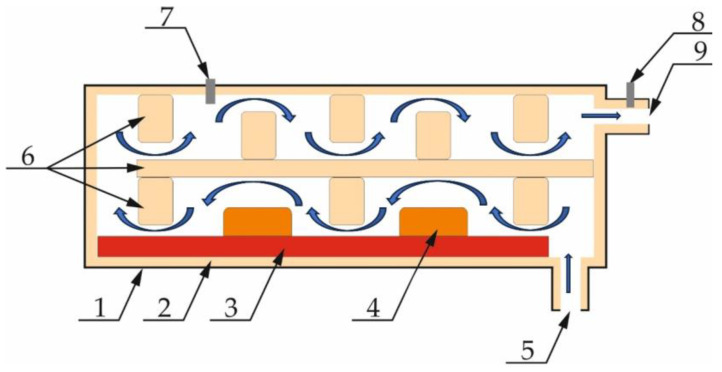
Illustrative diagram of the flue gas afterburning chamber: (1) steel armour; (2) insulation material; (3) heating elements; (4) ceramic mass fittings; (5) flue gas inlet; (6) insulation material fittings; (7) temperature sensor; (8) flue gas intake area; (9) flue gas outlet.

In the test rig, the flue gas afterburning reactor was placed downstream of the thermal regeneration gas outlet (Figure 8), which is also the flue gas inlet (5) to the thermal reactor (Figure 9).

The afterburning chamber (Figure 9) is the author’s solution, designed based on literature knowledge and experience. The outer shell (1) was made of structural steel with increased heat resistance. The interior of the walls was lined with a flexible insulating material made of biodegradable ceramic fibres, over which another insulating layer made of thick isostatically pressed ceramic plates (2) was applied. Furthermore, specially designed ceramic mouldings (4) were placed in the exhaust gas afterburning chamber space. Their function is to accumulate heat to limit the temperature drop in the afterburning chamber and, together with the insulating material, shape a specific flue gas flow path (6). The labyrinthine flue gas flow is designed to extend the flue gas path and residence time in the afterburner chamber, creating the right conditions for the combustion of the gases from the thermal regenerator chamber. A schematic diagram of the afterburner chamber is shown in Figure 9. The heating elements are a set of heaters in the form of Kanthal-type resistance coils with a total power of 30 kW, placed in chamotte shapes (3). A K-type thermocouple (7) was fed into the afterburner chamber, while flue gas sampling ports (8) were fitted after the flue gas outlet (9) from the afterburner chamber and before the filter system. The volume of the afterburning chamber of the flue gas is 1.2 m^3^. After heating, a temperature of 1000 °C ± 10 °C was maintained in the afterburning chamber of the flue gas. The created reactor does not interfere in any way with the design of the thermal regenerator. The measurement of electricity consumption is read by means of a connected metre (only the electricity consumption of the flue gas afterburning chamber).

In the industrial unit, the temperature of 600 °C was the lowest achievable temperature; therefore, it was slightly higher than the temperature required for the spent furan binder, which, according to the tests presented, was 521 °C.

Consequently, a temperature of 600 °C was used during the tests, and the regeneration (bed movement) was 30 min.

**Figure 10:**
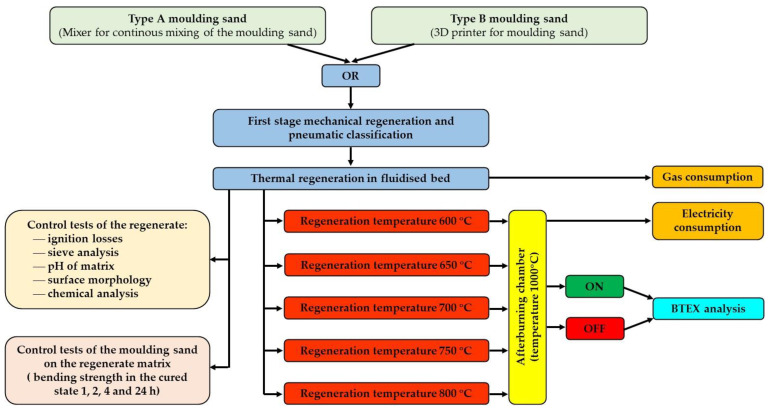
Flowchart of the research program.

The set-up of the bench was used to carry out the tests according to the programme shown in Figure 10.

## 3. Results

According to the adopted test scheme shown in Figure 10, the tests were carried out on the grain matrix and the moulding sand according to the criteria described in Table 3 for the different treatments applied to the two types of sand tested (sand made in a type A mixer for continuous mixing—Table 4—and sand from a type B 3D printer—Table 5). All regenerate samples (for each temperature range and sand type, the afterburning chamber was switched on or off) obtained from the thermal regenerator were taken 30 min after the start of the regenerate feeding after the mechanical treatment in the first stage. The regeneration dose to burn out the spent binder followed after the temperature in the regenerator chamber had stabilised.

In the first stage of testing, the thermal regeneration process was carried out using the standard design solution (with the afterburning chamber turned off). The temperature in the regenerator chamber was controlled in the range of 600–800 °C in 50 °C increments.

**Figure 11:**
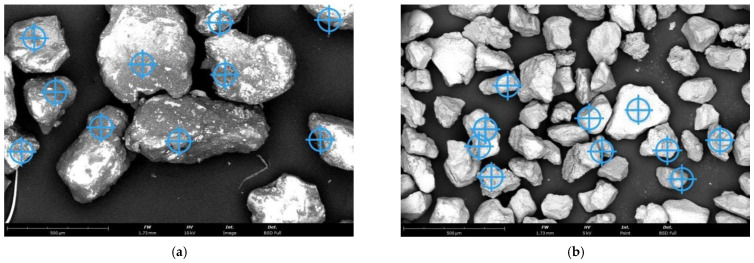
SEM image of grain matrixes after mechanical regeneration of the first stage: (**a**) type A moulding sand, mag. ×300; (**b**) type B moulding sand, mag. ×300.

**Figure 12:**
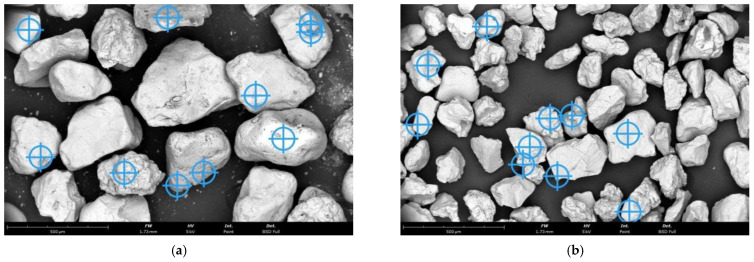
SEM image of grain matrixes after thermal regeneration at 600 °C: (**a**) type A moulding sand, mag. ×300; (**b**) type B moulding sand, mag. ×300.

**Figure 13:**
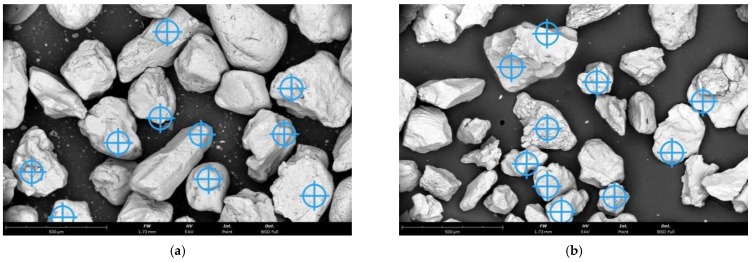
SEM image of grain matrixes after thermal regeneration at 800 °C: (**a**) type A moulding sand, mag. ×300; (**b**) type B moulding sand, mag. ×300.

The regenerate that was extracted after a given treatment was analysed in terms of its morphology and the residues of the spent binder on the surface. Table 6 shows the results of the chemical analysis carried out on the grain matrix using a scanning microscope with an EDS attachment following the realised treatment of the spent sand under different conditions (first-stage mechanical regeneration and thermal regeneration carried out in the assumed range of temperature changes). Figure 11 shows the granular matrix after the first stage of mechanical regeneration, Figure 12 after thermal regeneration at 600 °C, Figure 13 for the highest regeneration temperature of 800 °C, with the measurement locations marked as an example of the chemical analysis performed for all granular matrices subjected to this test, the corresponding results of which are shown in Table 6.

The main objective of the study was to check the effect of thermal regeneration on the quality of the matrix obtained and the sand prepared on it, as well as to determine the changes that occur with a decrease in regeneration temperature. For reasons of technological confidentiality, the recipe (amount of resin, hardener, and fresh sand) of the moulding sand used in the individual tests was not given. Both the input mass and the individual moulding sands prepared from the regenerates obtained were made with the same amount of resin and hardener added.

**Figure 14:**
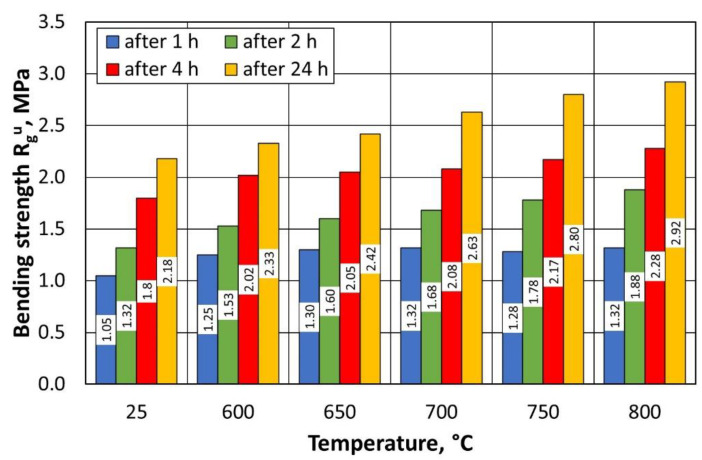
Bending strengths of type A moulding sand prepared on a regenerate matrix.

Several observations have been made on the basis of the results obtained from the tests of regenerates and moulding sand of the sand prepared on the basis of grain matrixes after the regeneration of A-type sand. As the temperature of the thermal regeneration process increases, the bending strength of the moulding sand increases R_g_^u^, which is due to the fact that the matrix grains are better cleaned of the remaining binder at a higher temperature (Figure 14). The value of the bending strength R_g_^u^ after 4 h is important for the technological process implemented in most companies. This is due to the production cycle, in which, in most cases, the prepared moulds—including the cores—are flooded with liquid metal after this time. As shown in Figure 14, there is little difference in the values between 600 °C and 800 °C, indicating an acceptable bending strength of the moulding sand for the production process, when the regeneration temperature is reduced by 200 °C.

The value of ignition losses decreases significantly (by a factor of six) when comparing the results after thermal regeneration at 600 °C versus first-stage mechanical regeneration. Furthermore, a similar range of ignition losses was recorded for material samples after thermal regeneration in the temperature range from 600 °C to 800 °C. The level of ignition loss values is much lower, indicating that the spent binder also burns effectively at lower temperatures and in a significant amount relative to the starting material, for which the ignition loss was 3.84% (Table 4).

**Figure 15:**
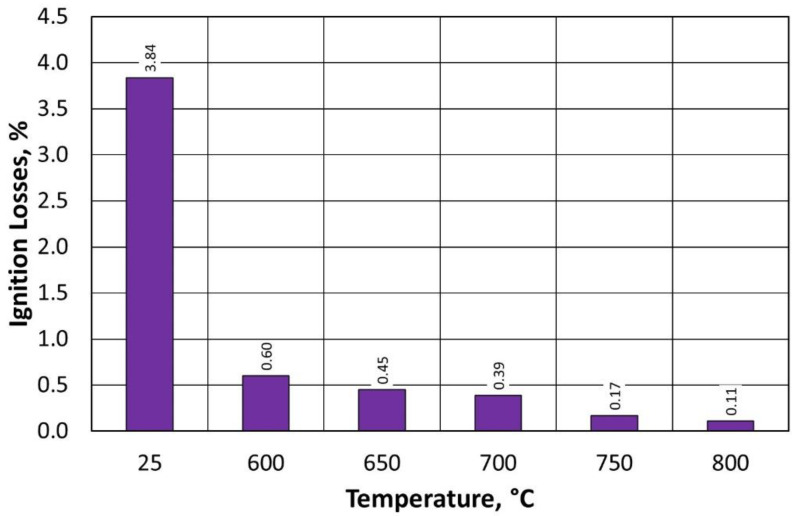
Regenerate ignition losses, depending on the temperature of regeneration and the treatment of the type A moulding sand.

In the investigated thermal regeneration temperature range, the parameters describing the size of the grain matrix change slightly (Figure 15). This means that small residues of the unfired binder accumulate in the irregularities on the surface of the matrix grains, as indicated by the ignition losses obtained.

**Figure 16:**
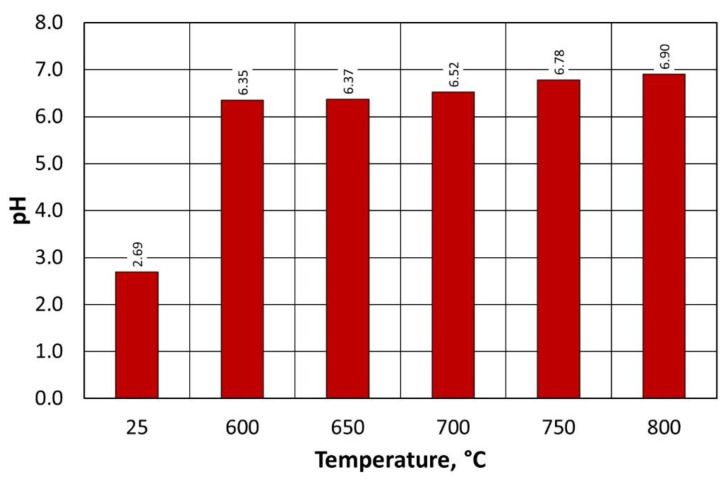
The pH of the regenerate depending on the temperature of regeneration and the treatment of the type A moulding sand.

The pH of the regenerate on the pH scale (Figure 16) varied between 6.35 and 6.90 over the range of the regeneration temperatures investigated; moreover, as the process temperature decreased, its pH had a lower value (more acidic). This is due to the small amounts of binder remaining on the quartz matrix, as indicated by the ignition losses. At the same time, irrespective of the regeneration temperature used, the resulting grain matrix had a pH closer to neutral than the regenerate after mechanical regeneration stage I (2.69).

**Figure 17:**
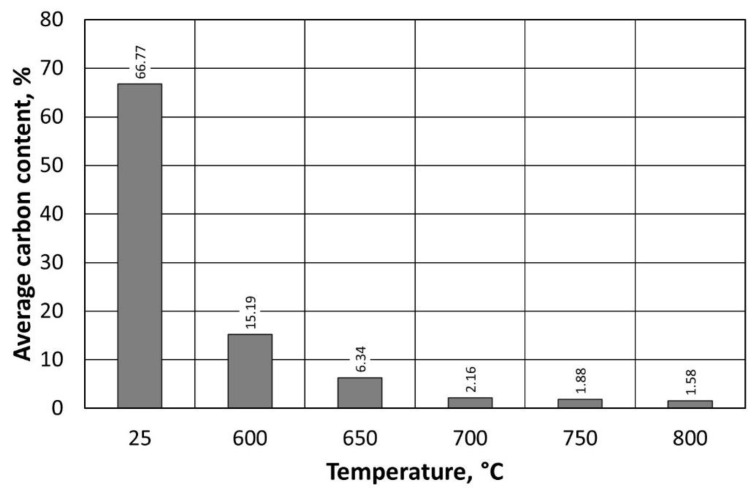
Carbon content on the surface of the regenerate grain matrix, depending on the temperature of regeneration and the treatment of the type A moulding sand.

Figure 17 shows the observed dependence of the change in carbon content with respect to temperature in the thermal regenerator chamber. In the case of the regenerate obtained at 600 °C, a significant increase in carbon and sulphur content is evident (Table 6), especially in relation to the regenerate obtained at 800 °C. Despite a reduction in the regeneration temperature by 200 °C, the amount remaining on the grain surface is very low. The residual sulphur content (from the sulphuric acid hardener) in the regenerate, in the range from 0.24% to 0.60%, has little effect on the reduction in the pH of the regenerate. In industrial production, this acidic character is often taken into account because it affects the kinetics of the moulding of the sand, requiring adjustments to the formulation of subsequent batches of moulding and core sand.

The data shown in Figure 15 and Figure 17 indicate that, with increasing temperature in the regenerator chamber, the amount of carbon remaining on the grain matrix decreases, which is related to the amount of burnt spent binder. This coincides with the change in the value of the ignition losses, which decreases with an increase in the burns of the spent binder at the higher temperature used in the regenerator chamber.

**Figure 18:**
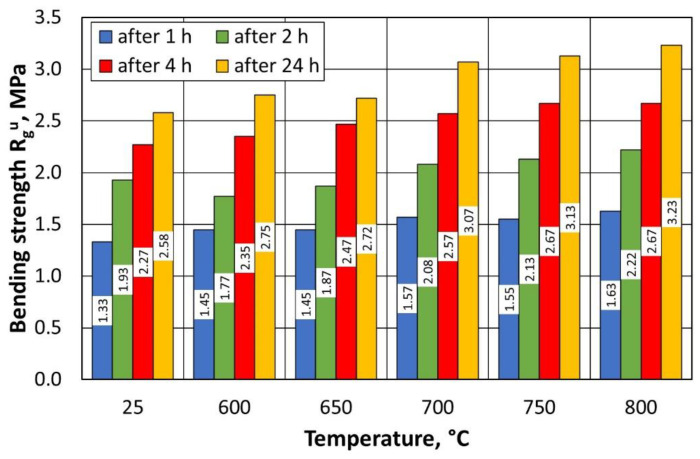
Bending strengths of type B moulding sand prepared on a regenerate matrix.

Several correlations can be observed from the summarised results in Table 5 for regenerates prepared from the type B moulding sand. Analogously to type A sand, as the temperature of the thermal regeneration process increases, the bending strength of R_g_^u^ in the moulding sand increases. This is due to the better purification of the matrix grains from the remaining binder at a higher temperature (Figure 18). The bending strength of the moulding sand on the regenerate matrix for 3D printing is higher than that for the type A moulding sand. This result is influenced by the greater homogeneity of the quartz matrix and the moulding sand preparation method. For type B moulding sand, the analysed strength in the tested regeneration temperature range changes by 0.42 MPa for the previously indicated curing time of 4 h.

**Figure 19:**
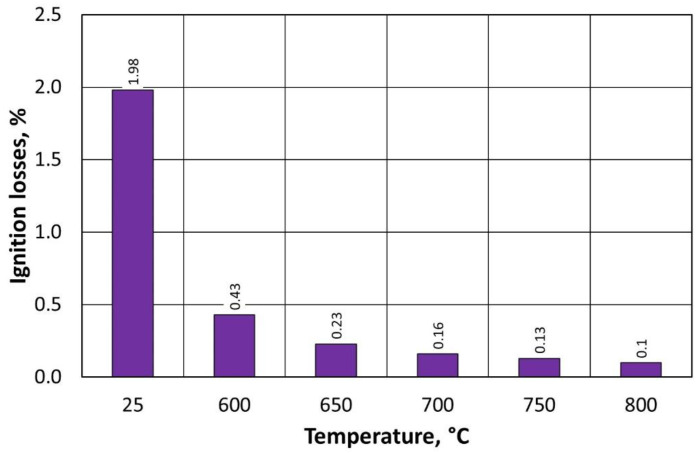
Regenerate ignition losses, depending on the temperature of regeneration and the treatment of the type B moulding sand.

The value of ignition losses decreases significantly (Figure 19) with a higher temperature change in the process carried out in the regenerator chamber. Differences between individual ignition losses values are small in the investigated process range, and the final value of the investigated parameter at 600 °C for type B moulding sand is lower than for type A moulding sand. This confirms that a lower input amount of binder on the grain matrix, after the first-stage regeneration, for the given conditions of the treatment carried out (time), more effectively cleanses the matrix grains of spent bound furfuryl resin. For the same regeneration time, the burning of a smaller amount of binder is more effective.

**Figure 20:**
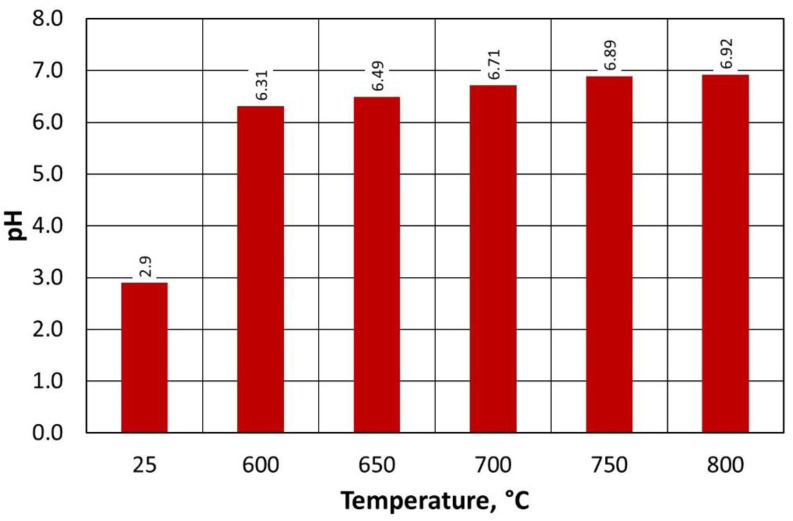
The pH of the regenerate, depending on the temperature of regeneration and the treatment of the type B moulding sand.

The pH values of the regenerate on the pH scale of type B moulding sand (Figure 20) are very similar to the determinations for type A moulding sand (Figure 14). This indicates the similar nature of the changes in acidity of the regenerate in relation to the set process temperature in the regenerator chamber. Despite the significantly different technologies for preparing the tested moulding sand (mixer for continuous mixing and 3D printer), the binder burning efficiency and the quality of the regenerate for each temperature are comparable.

**Figure 21:**
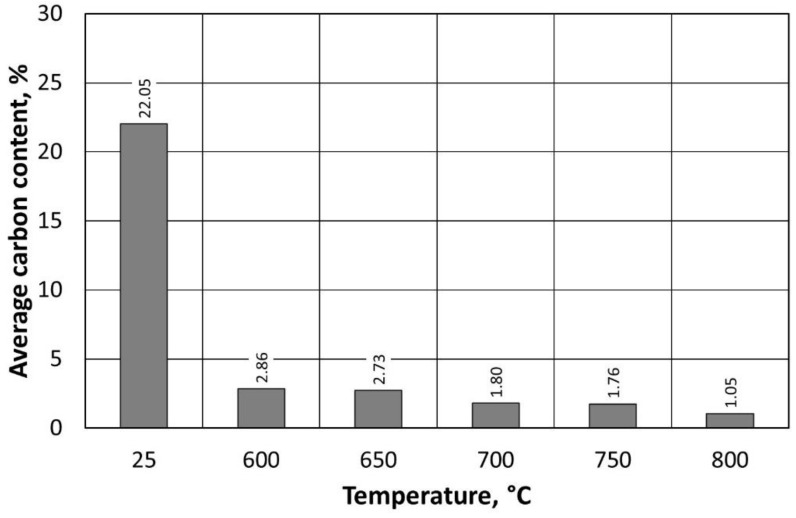
Carbon content on the surface of the regenerate grain matrix, depending on the temperature of regeneration and the treatment of the type B moulding sand.

The summarised data in Table 6 show a decrease in carbon content when an increase in thermal regeneration temperature is applied. The extent of these changes is shown in Figure 21. The greatest change in the amount of carbon remaining on the surface of the grain is observed between the sand spent after stage I regeneration and thermal regeneration at 600 °C.

**Figure 22:**
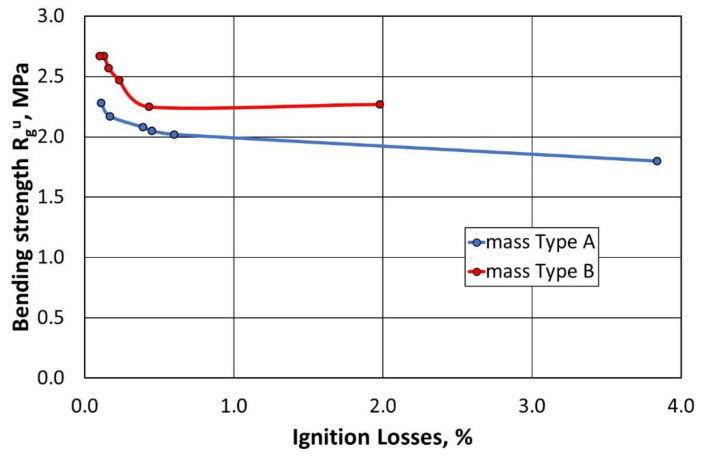
The bending strengths of the type A and B moulding sands as a function of the ignition losses.

The tests were carried out on spent moulding sand prepared using the same organic binder. The moulding sand preparation technology (binder delivery method; type of matrix grain) influences the strength. Figure 22 shows the change in the flexural strength of the moulding sand after 4 h of curing, prepared on a regenerate matrix obtained after the same treatment of the type A and type B moulding sand, compared to the ignition losses recorded under the given conditions. It can be observed that, with an increase in the regeneration temperature, a higher bending strength of the moulding sand is obtained on the regenerate matrix. The level of this strength depends on the initial amount of binder in the treated moulding sand.

Emissions of benzene, toluene, and xylene were measured downstream of the afterburner chamber and upstream of the filter system. Samples were taken through the measuring ports installed on the extraction system. The sampling of each set regeneration temperature in the regenerator chamber was carried out 30 min after a stable temperature was reached in the chamber. At the same time, the dosing of the first-stage regenerant intended for thermal regeneration (for each type of moulding sand A and B) was started. The concentration values of the individual gases shown in Table 7 are the arithmetic mean of the two measurements. Samples were taken and analysed using gas chromatography with flame ionisation detection (GC-FID) according to PN-EN 13649 [62].

**Figure 23:**
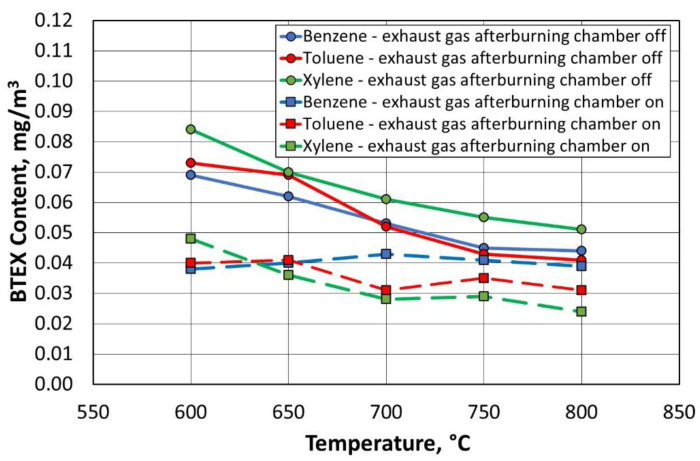
Concentrations of gases in the BTEX group for a given temperature of the thermal regeneration of type A moulding sand with the afterburner chamber on or off.

**Figure 24:**
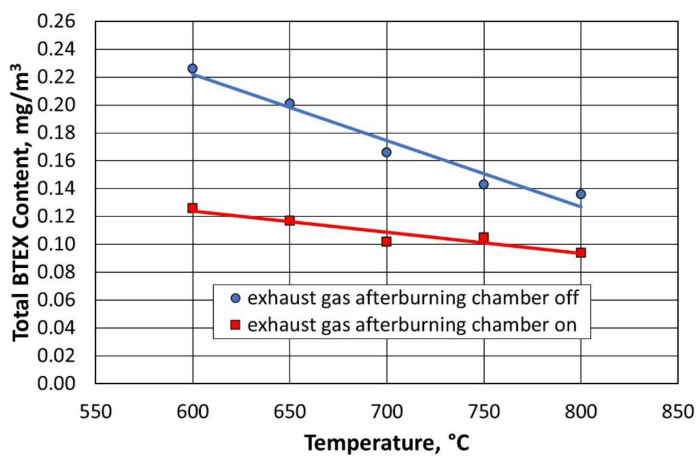
Total content of gases in the BTEX group for a given temperature of the thermal regeneration of type A moulding sand with the afterburner chamber on or off.

For all compounds from the BTEX group, the inclusion of the afterburning chamber reduced their emission to the environment, as shown in Figure 23 and Figure 24. Lowering the regeneration temperature of the moulding sand type A, with the afterburning chamber included, did not increase the release of individual compounds to the level recorded for the standard process (regeneration temperature of 800 °C, without afterburning chamber).

**Figure 25:**
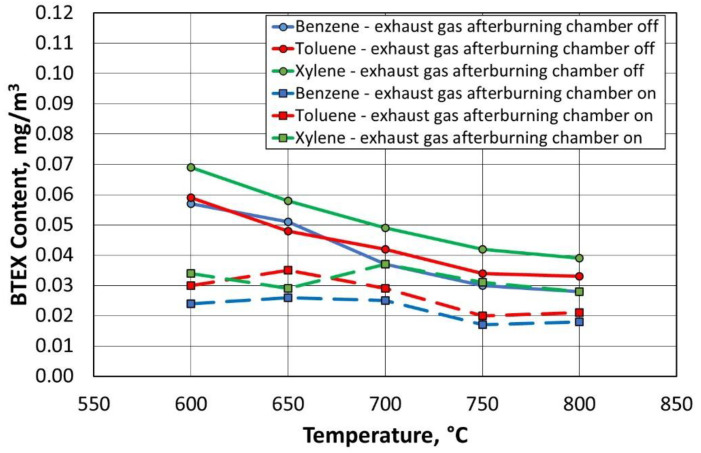
Concentrations of gases in the BTEX group for a given temperature of thermal regeneration of type B moulding sand with the afterburner chamber on or off.

**Figure 26:**
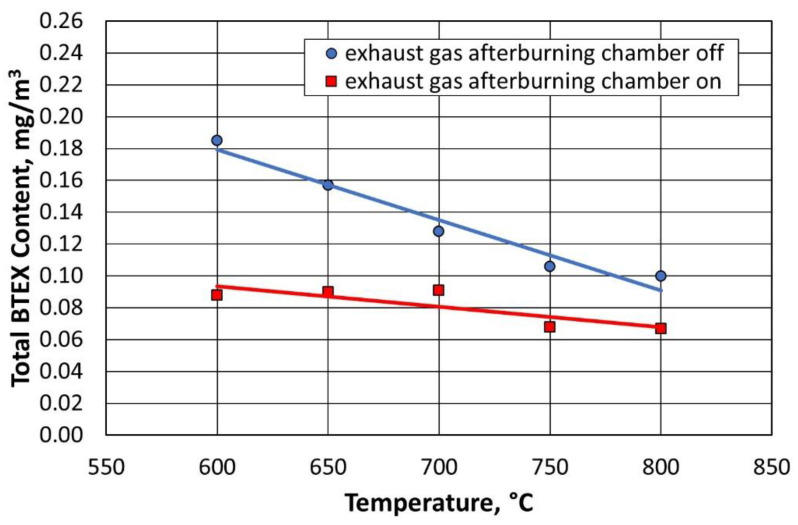
Total content of gases in the BTEX group for a given temperature of thermal regeneration of type B moulding sand with the afterburner chamber on or off.

Figure 25 and Figure 26 show the dependence of the change in concentration of the individual BTEX compounds for the type B moulding sand. It was observed that lowering the regeneration temperature and using a combustion chamber of flue gas afterburning did not increase the emission of harmful compounds into the environment.

Due to increasing issues in the field of the effects of air pollution on human health, BTEX testing is being carried out at various locations. BTEX gases such as benzene, toluene, and xylenes (meta-, ortho-, and para-) are found not only in the processes of the foundry industry and related industries (e.g., production of components for foundries) but also in many other areas, e.g., urban and everyday environments. This is due, among other things, to the significant impact of vehicle exhausts, aircraft engine gases, and the emission of a certain concentration of aromatic hydrocarbons into the air from paints or solvents, which are a source of BTEX [63,64,65].

The emissions from the thermal regenerator for comparison were related to other emission sources. The benzene concentration for the type B mass, with a regeneration temperature of 600 °C and the afterburning chamber turned on, reaches similar levels to the upper range of the measured benzene amounts for the remote rural areas [64]. Consecutively, the values are 0.024 mg/m^3^ (for the thermal regenerator) and 0.016 mg/m^3^ for the remote rural areas. On the other hand, for a regeneration temperature of 800 °C and the afterburning chamber turned on, the benzene value is almost at the same level (0.018 mg/m^3^).

Based on data from the literature [66,67,68] and the obtained data summarised in Table 7, it can be concluded that the amount of gases formed after the thermal regeneration process of the moulding masses under study, due to the afterburning chamber of the flue gas, is lower or at a level of concentration that is comparable to those in areas significantly distant from the zones where the sources of aromatic hydrocarbon emissions are located.

The measurement of the natural gas demand of the thermal regenerator was determined using a logger built into a standardised volume corrector. Measurement precision is ±0.2%. The standard natural gas demand for a 1 h period for regeneration at 800 °C averages 24.3 m^3^/h (arithmetic average of 3 h of measurement, excluding gas consumption needed for start-up). The recording of gas consumption started as soon as the temperature in the regenerator chamber stabilised and at the same time after the start of the dosing of the first-stage regenerant. Due to the different ambient temperatures and temperatures inside the regenerator chamber at the start of individual measurements, the natural gas demand during burner start-up and the time needed to reach the set temperature in the regenerator chamber were not taken into account. The gas consumption measurement was completed after 3 h of the thermal regeneration process.

The measurement of the electricity consumption of the afterburning chamber was determined using the electricity consumption meter. The accuracy of the measurement is ±1%. The measurement of the consumption of the afterburning chamber of the medium used to heat the flue gas was activated when a temperature of 1000 °C was reached inside the device, while a preset stable regeneration temperature was reached in the thermal regenerator chamber. The measurement time for electricity consumption was equal to the measurement time for gas consumption (3 h). Measurements taken during thermal regeneration of the type A moulding sand were used for the cost analysis.

The following prices for natural gas (*P_G_*) and electricity (*P_E_*), which were valid at the time of analysis preparation (01.2023 in Poland), were used for cost calculations:

- Natural gas: 0.58 EUR/m^3^;
- Electricity: 0.17 EUR/kWh.

Based on the data obtained on the average consumption of natural gas (*GC*) by the regenerator chamber and electricity (*EC*) by the exhaust gas postcombustion chamber during the thermal regeneration process, the economic aspect of the improved design solution used was assessed. The cost of the thermal regeneration operation (*WC_TR_*) was calculated according to Equation (5):

(5) $$WC_{TR}=GC\times P_{G}$$

On the other hand, the cost of the process with the afterburning chamber included (*WC_TR+AC_*), according to relation (6), is as follows:

(6) $$WC_{TR+AC}=\left(GC\times P_{G}\right)+\left(\mathrm{EC}\times P_{E}\right)$$

Table 8 summarises the data on the energy requirements of the thermal regenerator for different set temperatures in the regenerator chamber and with the afterburner chamber set as on and off. Calculating the operating cost of the thermal regenerator, according to Equations (5) and (6), does not take into account the energy required for the start-up of the burner and the time required to achieve the set temperature in the regenerator chamber. This cost is negligible with the continuous operation of the unit.

Based on calculations, it can be seen that the cost of 1 h of operation of a thermal regenerator with a flue gas afterburning chamber at 600 °C (at which the regenerate is of very good quality) is EUR 0.67/h cheaper than for a standard process carried out at 800 °C. This value seems small, but if this difference is taken into account on a yearly basis, for example, with an assumed operating time of the thermal regenerator of 24 h/day for 365 days, then an amount of EUR 5865.39 can be saved per year. The calculation only takes into account the cost of operating the unit. The costs of depreciation, possible breakdowns, or the repeated start-ups of the unit after it has been switched off are not taken into account.

**Figure 27:**
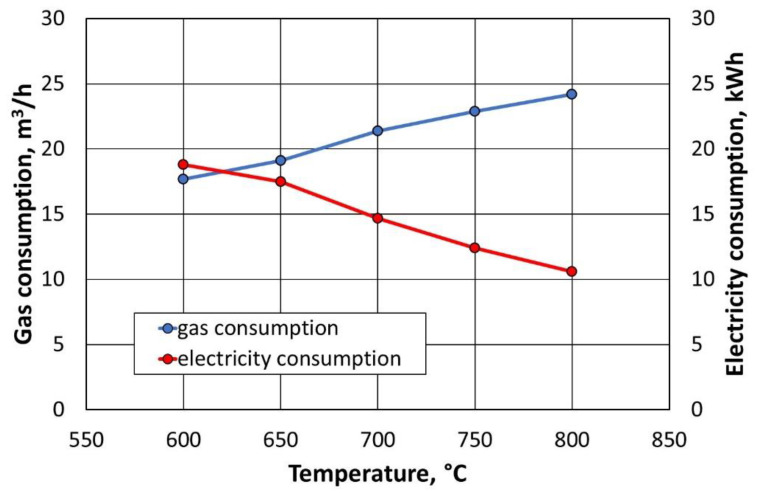
Consumption of natural gas and electricity depending on the temperature in the thermal regenerator chamber and the exhaust afterburning chamber turned on.

Figure 27 shows the variation in the consumption of natural gas and electricity depending on the regeneration temperature with the afterburning chamber switched on.

During the compilation of the energy demand data, it was found that, as the temperature in the regenerator chamber decreases, the power consumption of the afterburner chamber increases (Figure 27). This is due to the fact that, due to the lower temperature in the regenerator chamber (less gas consumption), the flue gas temperature is also lower. This results in less heat being supplied to the exhaust gas afterburning chamber. For this reason, the operating time of the heaters is longer, which generates increased energy consumption.

**Figure 28:**
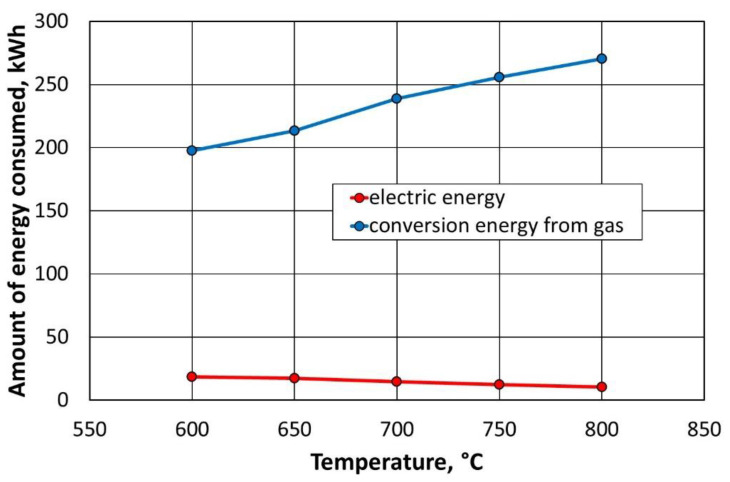
Energy consumption of the components of the thermal regeneration installation, depending on the temperature of the process.

For the amount of energy consumed by natural gas, a conversion factor equal to 11.17 kWh/m^3^ was introduced. This allows the comparison of the media used in terms of the amount of energy required to carry out the process. Considering the conversion factor, Figure 28 summarises the amounts of energy consumed by the various components of the thermal regeneration installation.

Based on the data presented in Figure 26, it can be concluded that the use of an additional gas combustion chamber generates 10 times less energy consumption, with respect to how much is consumed by the thermal regenerator chamber alone. At the same time, lowering the temperature in the regenerator slightly increases the afterburning chamber of the energy consumption of the flue gas. On the contrary, the energy consumption of natural gas decreases significantly when the regeneration temperature is reduced by 200 °C.

**Figure 29:**
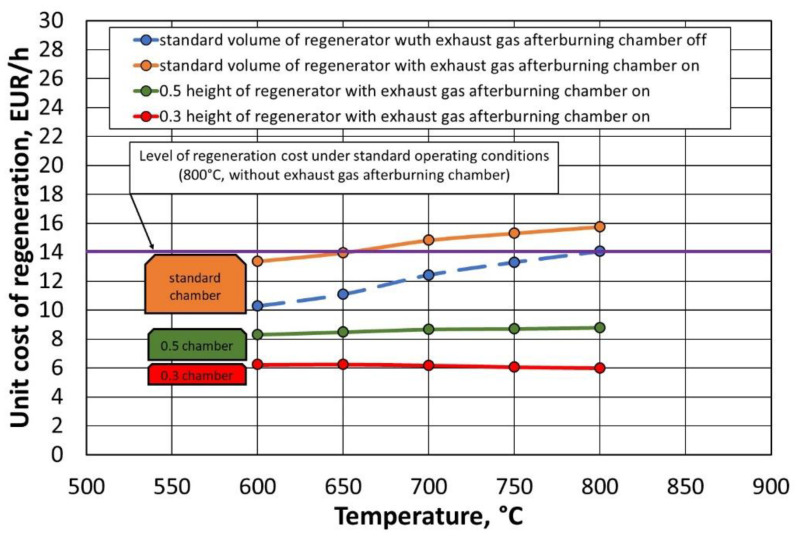
Summary of the unit cost of operation of a thermal regenerator with different heights of the regenerator chamber depending on the operating temperature for an on and off afterburner chamber.

In the search for a reduction in the cost of thermal regeneration, and while maintaining a comparable environmental impact, the issue of the size of the regenerator chamber is also important. A standard unit has a large volume. Under the assumption that a larger space to be heated requires the provision of more energy, in a situation of height reduction, we reduce the space that absorbs a certain amount of energy. The function of the previous high chamber is taken over by an additional afterburning chamber. From the point of view of device performance, the width and length of the thermal regenerator remain unchanged. Table 9 shows a simulation of the costs associated with reducing the height of the thermal regenerator chamber by 50% and 70%. The presented analysis applies to the situation of the solution of the regenerator design shown in Figure 7b (suggested solution). Figure 29 shows a graphical illustration of the estimated unit costs of the thermal regenerator operation performed.

Changing the height of the thermal regenerator chamber results in a significant cost reduction, especially between the standard solution and when the chamber is halved. Reducing the height by another 20% no longer results in such a significant decrease in costs, as the cost components of the afterburning chamber operation begin to play an important role.

In the analysis performed, the estimates of the energy requirements of the thermal regenerator were carried out by assuming, respectively, 50% and 30% of the height of the regenerator chamber relative to the standard solution (red and green lines in Figure 29). Based on the estimates (assuming proportional gas consumption depends on the size and height of the regenerator working chamber), the energy consumption of the thermal regenerator can be significantly reduced, which translates into a significant reduction in the unit cost of the thermal regenerator operation. By reducing up to 30% of the currently existing unit design, an amount of approximately EUR 68,480 can be saved annually.

## 4. Conclusions

Thermal regeneration studies have shown that the use of a flue gas afterburning chamber, as an improvement to an existing plant, has a beneficial effect on the process in economic and ecological terms.

On the basis of the realised research, it was found that the thermal regeneration process can be made more economical and ecological using a lower-than-standard regeneration temperature.

Due to the use of an afterburning chamber, the concentration of BTEX gases was reduced for both the standard thermal regeneration process performed at 800 °C and for the procedure performed at a reduced temperature of 600 °C.

The performance of the sieve analysis made it possible to show that the thermal regeneration process has a significantly lower impact on the degradation of the quartz matrix than in the case of the classic first-stage regeneration. This has an impact not only on the economic aspect but also on the ecological aspect because a smaller amount of quartz matrix is destroyed and discarded to a spent mass, or is extracted as dust in the fluidisation chamber.

The study showed that the cost of the thermal regeneration process, for a lower treatment temperature and the introduction of an additional power consumption component (the afterburning chamber) into the thermal regeneration installation, was reduced.

The cost of heating the afterburning chamber in which the flue gas is disposed of, in general, is lower than heating a regenerator chamber with a much larger capacity to a high temperature.

It was found on the basis of control studies that, by lowering the temperature inside the regenerator chamber, it is possible to obtain a regenerate with satisfactory quality parameters that is sufficient for the implementation of the production technology under the given foundry conditions.

Based on the estimates made, it was concluded that an additional reduction in the regenerator capacity of the working chamber and the use of a flue gas afterburning device can significantly reduce the demand for natural gas in the proposed plant and reduce the energy intensity of the process, thereby reducing the cost of thermal regeneration.

## Figures and Tables

**Table 1:** Results of the sieve analysis of the quartz sands used.

Parameter	Fresh Quartz Matri Type A	Fresh Quartz Matri Type B
d_a_—Arithmetic mean of grains [mm]	0.33	0.14
D_50_—Average grain size [mm]	0.31	0.13
F_g_—Main fraction [%]	91.96	97.07
GG—Degree of homogeneity [%]	75.00	93.00

**Table 2:** Parameters of the resin and hardener used in the moulding sand.

Properties and Chemical Composition	Type A		Type B	
	Binder	Hardener	Binder	Hardener
pH at 20 °C	6.5–7.5	<1	6–8	<1
Density at 20 °C	1.13–1.18 g/cm^3^	1.25–1.27 g/cm^3^	1.1–1.2 g/cm^3^	1.2–1.3 g/cm^3^
Ignition temperature	75 °C	>100 °C	66 °C	>100 °C
Furfuryl alcohol (CAS: 98-00-0)	>50%	N/D	60–100%	N/D
Phenol (CAS: 108-95-2)	<1%	N/D	N/D	N/D
Formaldehyde (CAS: 50-00-0)	≤0.09%	N/D	N/D	N/D
1,3-benzenediol (CAS: 108-46-3)	N/D	N/D	1–5%	N/D
3-aminopropyltriethoxysilane (CAS: 919-30-2)	N/D	N/D	<1%	N/D
P-toluene sulfonic acid (CAS: 104-15-4)	N/D	30–40%	N/D	60–100%
Xylensulfonic acid	N/D	30–40%	N/D	N/D
Sulphuric acid (CAS: 7664-93-9)	N/D	<3%	N/D	<5%

N/D—not designated.

**Table 3:** Proposed methods for evaluating the regeneration of different types of spent sand (according to the decreasing order of importance in a rating from 1 to 5).

Recommended Method for Evaluating the Regenerate	Type of Binder Material in the Spent Sand for Recovery of the Matrix				
	Synthetic Resin	Bentonite	Waterglass Curing		Cement
			CO_2_	Esters	
Ignition losses	(1)	(3)	(-)	(-)	(-)
Bending strength	(2)	(1)	(2)	(2)	(1)
pH value	(3)	(-)	(4)	(5)	(3)
Sieve analysis	(4)	(4)	(3)	(3)	(4)
Surface morphology	(5)	(5)	(5)	(5)	(5)
Binder volume	(-)	(2)	(-)	(-)	(2)
Binder activity	(-)	(3)	(-)	(-)	(-)
Content Na_2_O	(-)	(-)	(1)	(1)	(-)

**Table 4:** Parameters of the grain matrix and the moulding sand made on this grain matrix for type A moulding sand.

Parameters		First-Stage Mechanical Regeneration	Regeneration Temperature				
			600 °C	650 °C	700 °C	750 °C	800 °C
Ignition losses [%]		3.84	0.60	0.45	0.39	0.17	0.11
Bending strength R_g_^u^ [MPa]	1 [h]	1.05	1.25	1.30	1.32	1.28	1.32
	2 [h]	1.32	1.53	1.60	1.68	1.78	1.88
	4 [h]	1.80	2.02	2.05	2.08	2.17	2.28
	24 [h]	2.18	2.33	2.42	2.63	2.80	2.92
Chemical pH		2.69	6.35	6.37	6.52	6.78	6.90
Sieve analysis	d_a_ [mm]	0.28	0.26	0.26	0.26	0.27	0.28
	D_50_ [mm]	0.24	0.23	0.23	0.23	0.23	0.23
	F_g_ [%]	85.65	84.02	86.92	89.69	89.73	92.13
	GG [%]	70.00	71.00	74.00	77.00	77.00	77.00

**Table 5:** Parameters of the grain matrix and the moulding sand made on this grain matrix for type B moulding sand.

Parameters		First-Stage Mechanical Regeneration	Regeneration Temperature				
			600 °C	650 °C	700 °C	750 °C	800 °C
Ignition losses [%]		1.98	0.43	0.23	0.16	0.13	0.10
Bending strength R_g_^u^ [MPa]	1 [h]	1.33	1.45	1.45	1.57	1.55	1.63
	2 [h]	1.93	1.77	1.87	2.08	2.13	2.22
	4 [h]	2.27	2.25	2.47	2.57	2.67	2.67
	24 [h]	2.58	2.75	2.72	3.07	3.13	3.23
Chemical pH		2.90	6.31	6.49	6.71	6.89	6.92
Sieve analysis	d_a_ [mm]	0.13	0.15	0.15	0.16	0.15	0.15
	D_50_ [mm]	0.13	0.14	0.14	0.14	0.13	0.13
	F_g_ [%]	97.37	96.11	95.82	96.27	96.97	96.82
	GG [%]	93.00	90.00	90.00	89.00	91.00	93.00

**Table 6:** Chemical composition determined on the grain surface using scanning microscopy with EDS attachment of type A and B moulding sand.

Type of Treatment	Moulding Sand	Type A					Type B				
	Parameters	C	O	Al	Si	S	C	O	Al	Si	S
1st stage mechanical regeneration	Max [%]	77.29	41.72	1.56	12.17	4.53	47.35	69.00	9.20	45.23	2.06
	Min [%]	44.33	19.90	0.00	1.26	0.65	5.33	30.83	0.00	9.85	0.25
	Average [%]	66.77	27.04	0.44	3.71	2.05	22.05	50.60	1.50	24.91	0.94
	Standard Deviation σ	8.90	6.80	0.44	3.21	1.15	13.22	12.16	2.76	11.85	0.70
Thermal regeneration 600 °C	Max [%]	66.53	76.16	3.00	35.48	1.19	15.05	73.81	1.79	33.16	0.42
	Min [%]	0.81	28.99	0.33	2.25	0.26	0.00	62.71	0.26	21.51	0.00
	Average [%]	15.19	59.61	1.03	23.58	0.60	2.86	69.47	0.60	26.81	0.25
	Standard Deviation σ	20.65	12.40	0.76	11.20	0.29	4.53	3.48	0.48	3.60	0.15
Thermal regeneration 650 °C	Max [%]	16.78	74.02	9.17	29.02	0.60	4.96	74.68	0.39	44.67	0.66
	Min [%]	0.81	57.82	0.00	15.66	0.00	0.20	49.85	0.14	23.11	0.28
	Average [%]	6.34	68.41	1.74	23.15	0.37	2.73	65.85	0.33	30.67	0.42
	Standard Deviation σ	5.75	4.75	2.67	4.91	0.22	1.67	7.76	0.08	6.89	0.15
Thermal regeneration 700 °C	Max [%]	3.56	70.07	4.13	75.81	0.70	3.18	72.61	2.83	76.09	0.55
	Min [%]	0.81	22.07	0.33	25.37	0.28	0.80	17.49	0.36	23.35	0.00
	Average [%]	2.16	57.54	1.35	38.50	0.45	1.80	61.54	0.91	35.49	0.26
	Standard Deviation σ	0.93	15.16	1.20	15.34	0.17	0.73	16.80	0.87	15.78	0.23
Thermal regeneration 750 °C	Max [%]	3.57	71.25	5.68	72.45	1.60	3.99	73.99	0.77	40.07	0.47
	Min [%]	0.00	19.20	0.36	23.95	0.00	0.00	57.53	0.18	25.35	0.11
	Average [%]	1.88	58.09	1.57	38.08	0.38	1.76	66.35	0.49	31.14	0.27
	Standard Deviation σ	1.07	16.06	2.03	14.65	0.45	1.09	4.86	0.20	4.93	0.11
Thermal regeneration 800 °C	Max [%]	3.10	75.95	3.24	71.33	0.32	2.58	75.05	23.54	65.23	0.41
	Min [%]	0.10	26.13	0.00	19.34	0.00	0.00	33.13	0.12	15.67	0.00
	Average [%]	1.58	63.33	0.98	33.87	0.24	1.05	60.38	4.61	33.76	0.20
	Standard Deviation σ	1.10	14.12	1.02	14.49	0.13	0.81	15.04	7.68	15.95	0.18

**Table 7:** Summary of gas concentrations determined for the applied thermal regeneration temperature of type A and B moulding sands.

Temperature in the Thermal Regenerator Chamber [°C]	Afterburner Chamber	Moulding Sand Type A			Moulding Sand Type B		
		Benzene [mg/m^3^]	Toluene [mg/m^3^]	Xylene [mg/m^3^]	Benzene [mg/m^3^]	Toluene [mg/m^3^]	Xylene [mg/m^3^]
600	OFF	0.069	0.073	0.084	0.057	0.059	0.069
	ON	0.038	0.04	0.048	0.024	0.03	0.034
650	OFF	0.062	0.069	0.070	0.051	0.048	0.058
	ON	0.040	0.041	0.036	0.026	0.035	0.029
700	OFF	0.053	0.052	0.061	0.037	0.042	0.049
	ON	0.043	0.031	0.028	0.025	0.029	0.037
750	OFF	0.045	0.043	0.055	0.030	0.034	0.042
	ON	0.041	0.035	0.029	0.017	0.02	0.031
800	OFF	0.044	0.041	0.051	0.028	0.033	0.039
	ON	0.039	0.031	0.024	0.018	0.021	0.028

**Table 8:** Electricity consumption, natural gas consumption, and unit cost of thermal regenerator operation for different set temperatures in the thermal regenerator.

Temperature in the Thermal Regenerator Chamber [°C]	Afterburner Chamber	Natural Gas Consumption [m^3^/h]	Natural Gas Consumption [kWh]	Electricity Consumption [kWh]	Cost per Hour of Operation of the Thermal Regenerator [EUR/h]
600	OFF	17.80	198.83	—	10.29
	ON	17.70	197.71	18.80	13.38
650	OFF	19.20	214.46	—	11.10
	ON	19.10	213.35	17.50	13.97
700	OFF	21.50	240.16	—	12.43
	ON	21.40	239.04	14.70	14.83
750	OFF	23.00	256.91	—	13.30
	ON	22.90	255.79	12.40	15.32
800	OFF	24.30	271.43	—	14.05
	ON	24.20	270.31	10.60	15.77

**Table 9:** Unit cost of the operation of a thermal regenerator of different heights and for different set temperatures in the regenerator chamber.

Temperature in the Thermal Regenerator Chamber [°C]	Afterburner Chamber	Cost of Working 1 h of Standard Height Thermal Regenerator [EUR/h]	Cost of Working 1 h of Thermal Regenerator 0.5 Standard Height [EUR/h]	Cost of Working 1 h of Thermal Regenerator 0.3 Standard Height [EUR/h]
600	OFF	10.29	—	—
	ON	13.38	8.29	6.23
650	OFF	11.10	—	—
	ON	13.97	8.48	6.26
700	OFF	12.43	—	—
	ON	14.83	8.68	6.19
750	OFF	13.30	—	—
	ON	15.32	8.73	6.07
800	OFF	14.05	—	—
	ON	15.77	8.80	5.99

## Data Availability

The data that support the findings of this study are available from the corresponding authors (M.Ł.) upon reasonable request.

## References

[1] Dańko J., Dańko R., Łucarz M. (2007). Processes and Cevices for Reclamation of Used Moulding Sands.

[2] Eun Y.K., Kyeong H.K., Jae H.B., Inseong H., Man S.L. (2021). Wet regeneration of waste artificial sand used in sand casting using chemical solutions. Environ. Eng. Res..

[3] Fan Z., Huang N.Y., Dong X.P. (2004). In house reuse and reclamation of used foundry sands with sodium silicate binder. Int. J. Cast Met. Res..

[4] Wang L.C., Jiang W.M., Gong X.L., Liu F.C., Fan Z.T. (2019). Recycling water glass from wet reclamation sewage of waste sodium silicate-bonded sand. China Foundry.

[5] Kayal S., Chakrabarti B.K. (2008). Reclamation and utilisation of foundry waste sand. High Temp. Mater..

[6] Zanetti M.C., Fiore S. (2003). Foundry processes: The recovery of green moulding sands for core operations. Resour. Conserv. Recycl..

[7] Zanetti M.C., Fiore S. (2008). Industrial treatment processes for recycling of green foundry sands. Int. J. Cast Met. Res..

[8] Dańko J., Dańko R., Holtzer M. (2003). Reclamation of used sands in foundry production. Metalurgija.

[9] Mitterpach J., Hroncová E., Ladomerský J., Balco K. (2017). Environmental analysis of waste foundry sand via life cycle assessment. Environ. Sci. Pollut. Res..

[10] Dańko R., Jezierski J., Holtzer M. (2016). Physical and chemical characteristics of after-reclamation dust from used sand moulds. Arab. J. Geosci..

[11] Silva E.C., Masiero I., Guesser W.L. (2020). Comparing sands from different reclamation processes for use in the core room of cylinder heads and cylinder blocks production. Int. J. Met..

[12] Zitian F., Fuchu L., Wei L., Guona L. (2014). A new low-cost method of reclaiming mixed foundry waste sand based on wet-thermal composite reclamation. China Foundry.

[13] Khan M.M., Mahajani S.M., Jadhav G.N., Vishwakarma R., Malgaonkar V., Mandre S. (2021). A multistakeholder approach and techno-economic analysis of a mechanical reclamation process for waste foundry sand in the Indian context. Resour. Conserv. Recycl..

[14] Khan M.M., Mahajani S.M., Jadhav G.N., Vishwakarma R., Malgaonkar V., Mandre S. (2021). Mechanical and thermal methods for reclamation of waste foundry sand. J. Environ. Manag..

[15] Khan M.M., Singh M., Mahajani S.M., Jadhav G.N., Mandre S. (2018). Reclamation of used green sand in small scale foundries. J. Mater. Process. Technol..

[16] Khan M.M., Singh M., Jadhav G.N., Mahajani S.M., Mandre S. (2020). Characterization of Waste and Reclaimed Green Sand Used in Foundry Processing. Silicon.

[17] Anwar N., Jalava K., Orkas J. (2022). Experimental study of inorganic foundry sand binders for mold and cast quality. Int. J. Met..

[18] Dańko R. (2012). Strength Model of Self-Setting Moulding Sands with Synthetic Resins in an Aspect of the Integrated Matrix Recycling Process.

[19] Łatas W., Dańko R., Czapla P. (2020). Application of 3-D Drucker–Prager Material Model to Determine Optimal Operating Parameters of Centrifugal Regeneration Device. Materials.

[20] Saripalli N.J., Sonawane D.R. (2018). Assessment of Reclaiming Process of Sand as Foundry Waste for Industrial Usage. Int. J. Sci. Manag. Stud..

[21] Nyembwe K.D., Kabasele J.K. (2022). Sustainability assessment of thermal and mechanical reclamation of foundry chromite sand. S. Afr. J. Ind. Eng..

[22] Cruz N., Briens C., Berruti F. (2009). Green sand reclamation using a fluidized bed with an attrition nozzle. Resour. Conserv. Recycl..

[23] Dańko R., Dańko J., Skrzyński M. (2017). Assessment of the possibility of using reclaimed materials for making cores by the blowing method. Arch. Found. Eng..

[24] Holtzer M., Dańko R., Kmita A., Drożyński D., Kubecki M., Skrzyński M., Roczniak A. (2020). Environmental impact of the reclaimed sand addition to molding sand with furan and phenol-formaldehyde resin—A comparison. Materials.

[25] Skrzyński M. (2020). Influence of the process treatment on the amount and grain structure of after reclamation dusts. Arch. Found. Eng..

[26] Skrzyński M., Dańko R. (2019). Primary used sand reclamation process efficiency. Arch. Found. Eng..

[27] Major-Gabryś K., Hosadyna-Kondracka M., Skrzyński M., Stachurek I. (2022). The influence of biomaterial in the binder composition on the quality of reclaim from furan no-bake sands. Arch. Civ. Eng..

[28] Chong-Lyuck P., Byoung-Gon K., Youngchul Y. (2012). The regeneration of waste foundry sand and residue stabilization using coal refuse. J. Hazard. Mater..

[29] Patange G.S., Khond M.P., Rathod H.J., Chhadva K.B. (2013). Investigation of foundry waste sand reclamation process for small and medium scale indian foundry. Int. J. Ind. Eng. Technol..

[30] Łucarz M. (2008). The effect of mechanical reclamation on the wear of silica sand grains. Metalurgija.

[31] Monish A., Krishna B.S.V.S.R. (2019). Optimization of time and temperature for thermal reclamation of furan resin based sand. J. Recent Technol. Eng..

[32] Rayjadhav S.B., Mhamane D.A., Shinde V.D. (2020). Assessment of sand reclamation techniques and sand quality in thermal reclamation. Int. J. Product. Qual. Manag..

[33] Severo J.A., Modolo R.C.E., Moraes C.A.M., Zinani F.S.F. (2018). Thermal regeneration of waste foundry phenolic sand in a lab scale fluidized bed. Matéria.

[34] Svidró J.T., Diószegi A., Svidró J., Ferenczi T. (2017). Thermophysical aspects of reclaimed moulding sand addition to the epoxy-SO2 coremaking system studied by Fourier thermal analysis. J. Therm. Anal. Calorim..

[35] Wan P., Zhou J., Li Y., Yin Y., Peng X., Ji X., Shen X. (2022). Kinetic analysis of resin binder for casting in combustion decomposition process. J. Therm. Anal. Calorim..

[36] Li Y.L., Wu G.H., Liu W.C., Chen A.T., Zhang L., Wang Y.X. (2017). Effect of reclaimed sand additions on mechanical properties and fracture behavior of furan no-bake resin sand. China Foundry.

[37] Saboura M.R., Akbaria M., Dezvareha G. (2020). Utilization of color change and image processing to evaluate the Waste Foundry Sand reclamation level. J. Mater. Res. Technol..

[38] Andrade R.M., Cava S., Silva S.N., Soledade L.E.B., Rossi C.C., Leite E.R., Paskocimas C.A., Varela J.A., Longo E. (2005). Foundry sand recycling in the troughs of blast furnaces: A technical note. J. Mater. Process..

[39] Kumar P., Gandhi N.M. (2020). Achieving environmental sustainability in the shell mould foundry through thermal reclamation. Trans. Can. Soc. Mech. Eng..

[40] Wang L.L., Liu Y., Pan L., Fang Y. (2018). Study on Regeneration Processing Technology of Used Pearl Coated Sand. Zhuzao/Foundry.

[41] Łucarz M. (2013). The influence of the configuration of operating parameters of a machine for thermal reclamation on the efficiency of reclamation process. Arch. Metall. Mater..

[42] Łucarz M. (2006). The condition of silica sand grains surface subjected to reclamation treatment. Metalurgija.

[43] Łucarz M. (2018). Theoretical Conditions of the Selection of the Thermal Reclamation Temperature of Moulding Sands with Organic Binders.

[44] Łucarz M., Dereń M. (2017). Conditions of Thermal Reclamation Process Realization on a Sample of Spent Moulding Sand from an Aluminum Alloy Foundry Plant. Arch. Found. Eng..

[45] Dereń M., Łucarz M., Roczniak A., Kmita A. (2017). Influence of Reclamation Process on the Ecological Quality of Reclaim Sand. Arch. Found. Eng..

[46] Krogulec N.J. (1994). Industrial waste incineration. Ochr. Powietrza I Probl. Odpad..

[47] Łucarz M. (2012). Ecological station for the thermal regeneration of spent moulding sand. Arch. Found. Eng..

[48] Mokrosz W. Ecological aspects of flue gas cleaning from municipal and industrial waste incineration plants. Proceedings of the Materiały z X konferencji “POL-EMIS 2010”.

[49] https://www.athon.com.pl.

[50] Paul P., Belhaj E., Diliberto C., Apedo K.L., Feugeas F. (2021). Comprehensive Characterization of Spent Chemical Foundry Sand for Use in Concrete. Sustainability.

[51] Cioli F., Abbà A., Alias C., Sorlini S. (2022). Reuse or Disposal of Waste Foundry Sand: An Insight into Environmental Aspects. Appl. Sci..

[52] Jonczy I., Kamińska M., Bilewska K., Gerle A. (2018). Crystalline Phases in the Waste Foundry Sands Based on Quartz Sand Matrix. Eng. Prot. Environ..

[53] Mizuki T., Kanno T. (2018). Establishment of Casting Manufacturing Technology by Introducing an Artificial Sand Mold with Furan Resin and Realizing a Clean Foundry. Int. J. Met..

[54] Bobrowski A., Kaczmarska K., Drożyński D., Woźniak F., Dereń M., Grabowska B., Żymankowska-Kumon S., Szucki M. (2023). 3D printed (binder jetting) furan molding and core sands—Thermal deformation, mechanical and technological properties. Materials.

[55] Bobrowski A., Woźniak F., Żymankowska-Kumon S., Kaczmarska K., Grabowska B., Dereń M., Żuchliński R. (2023). The influence of 3D printing core construction (binder jetting) on the amount of generated gases in the environmental and technological aspect. Materials.

[56] Dańko J., Łucarz M. (1995). Regenerative treatment and pneumatic classification of a spent moulding sand sample as a basis for assessing its suitability for mechanical regeneration. Solidif. Met. Alloys.

[57] (1983). Foundry Moulding Materials—Measurement of Strength.

[58] (2000). Inspection Sieves—Technical Requirements and Testing—Test Sieves of Metal Wire Cloth.

[59] Łucarz M. (1996). Development of the Design Basis of Centrifugal Moulding Sand Regenerators. Ph.D. Thesis.

[60] Rojek M. (2011). Methodology for diagnostic testing of layered polymer matrix composite materials. Open Access Libr..

[61] http://tinkeromega.com/category/thermal-reclamation-1.

[62] (2001). Stationary Source Emissions—Determination of Mass Concentration of Individual Gaseous Organic Compounds —Method Using Activated Carbon and Solvent Desorption.

[63] Leusch F., Bartkow M. (2010). A Short Primer on Benzene, Toluene, Ethylbenzene and Xylenes (BTEX) in the Environment and in Hydraulic Fracturing Fluids.

[64] US Department of Health and Human Services, Public Health Service (2005). Report on Carcinogens.

[65] (2008). Guidelines for Drinking Water Quality.

[66] Hamid H.H.A., Jumah N.S., Latif M.T., Kannan N. (2017). BTEXs in Indoor and Outdoor Air Samples: Source Apportionment and Health Risk Assessment of Benzene. Environ. Sci. Public Health.

[67] Chang C.T., Chen B.Y. (2008). Toxicity assessment of volatile organic compounds and polycyclic aromatichydrocarbons in motorcycle exhaust. J. Hazard Mater..

[68] Lan T.T.N., Binh N.T.T. (2012). Daily roadside BTEX concentrations in East Asia measured by the Lanwatsu, Radiello and Ultra I SKS passive samplers. Sci. Total Environ..

